# Letrozole-stimulated endometrial preparation protocol is a superior alternative to hormone replacement treatment for frozen embryo transfer in women with polycystic ovary syndrome, a cohort study

**DOI:** 10.1186/s12958-023-01154-x

**Published:** 2023-10-27

**Authors:** Xiaojuan Wang, Yuan Li, Hongzhuan Tan, Sufen Cai, Shujuan Ma, Yangqin Peng, Hui Guo, Xiaofeng Li, Yi Tang, Shunji Zhang, Ge Lin, Fei Gong

**Affiliations:** 1 Department of Epidemiology and Health Statistics, School of Public Health, Central South University, Changsha, 410008 Hunan China; 2 Clinical Research Center for Reproduction and Genetics in Hunan Province, Reproductive and Genetic Hospital of CITIC-XIANGYA, NO. 567 Tongzipo West Road, Yuelu District, Changsha city, 410008 Hunan Province China; 3 Laboratory of Reproductive and Stem Cell Engineering, Key Laboratory of National Health and Family Planning Commission, Central South University, Changsha, 410008 Hunan China

**Keywords:** Polycystic ovary syndrome, Frozen embryo transfer, Endometrial preparation, Letrozole, Hormone replacement treatment

## Abstract

**Background:**

The current routine endometrial preparation protocol for women with polycystic ovary syndrome (PCOS) is hormone replacement treatment (HRT). Letrozole is rarely used in frozen embryo cycles. Evidence confirming whether letrozole-stimulated (LS) protocol is suitable for frozen embryo transfer in patients with PCOS and for whom is suitable remains lacking.

**Methods:**

This was a retrospective cohort study involving all frozen embryo transfer cycles with LS and HRT for PCOS during the period from Jan 2019 to December 2020 at a tertiary care center. Multivariate Logistic regression was used to analyze the differences in clinical pregnancy rate, live birth rate, miscarriage rate, the incidence of other pregnancy and obstetric outcomes between LS and HRT protocols after adjusting for possible confounding factors. Subgroup analysis was used to explore the population for which LS protocol was suitable.

**Results:**

The results of multivariate logistic regression showed that LS was significantly associated with a higher clinical pregnancy rate (70.9% vs. 64.4%;aOR:1.41, 95%CI: 1.18,1.68), live birth rate (60.5% vs. 51.4% aOR:1.49, 95%CI: 1.27,1.76), and a lower risk of miscarriage (14.7% vs. 20.1% aOR: 0.68, 95%CI: 0.53,0.89), hypertensive disorders of pregnancy (6.7% vs. 8.9% aOR: 0.63, 95%CI: 0.42,0.95), and gestational diabetes mellitus (16.7% vs. 20.7% aOR:0.71, 95%CI: 0.53,0.93) than HRT. There were no significant differences in other outcomes such as preterm birth, cesarean delivery, small for gestational age, or large for gestational age between the two endometrial preparation protocols. Subgroup analysis showed that LS had higher live birth rates than HRT in most of the subgroups; in the three subgroups of maternal age ≥ 35 years, menstrual cycle < 35 days, and no insulin resistance, the live birth rates of the two endometrial preparation protocols were comparable.

**Conclusions:**

LS protocol could improve the live birth rate and reduce the incidence of miscarriage, hypertensive disorders of pregnancy and gestational diabetes mellitus in patients with PCOS. LS protocol is suitable for all types of patients with PCOS. LS should be considered the preferred endometrial preparation protocol for women with PCOS.

**Supplementary Information:**

The online version contains supplementary material available at 10.1186/s12958-023-01154-x.

## Background

Polycystic ovary syndrome (PCOS) is the most common endocrinopathy affecting reproductive-aged women, with heterogeneous features that include irregular or absent menstrual periods, hyperandrogenism, and related metabolic and.

psychological sequelae [1]. The prevalence of PCOS ranges from 5% to 18% depending on the population studied and definitions used [2]. PCOS is the primary cause of and accounts for 90% of anovulatory infertility [3]. Studies have found that frozen embryo transfer (FET) can lower the rates of pregnancy loss and ovarian hyperstimulation syndrome (OHSS) and improve the live birth rate (LBR) compared to fresh embryo transfer in women with PCOS [4]. FET is considered to be more suitable for infertile women with PCOS, and freeze-only strategy is recommended by International Evidence-based Guideline [2].

Endometrial preparation is the key to the success of FET. By far, no superiority has been shown for any regimen for endometrial preparation for women with PCOS. Since women with PCOS typically have irregular or oligo-anovulation [5], the most frequently used cycle regimen for women with PCOS is hormonal replacement treatment (HRT). In HRT cycles, the endometrium is prepared with programmed estrogen and progesterone, providing convenience for planning the time of embryo transfer. However, HRT is expensive. Furthermore, once pregnancy is established, exogenous estrogen and progesterone cannot be withdrawn until the placenta is formed to substitute for the absent corpus luteum. Recent findings suggest that the absence of the corpus luteum in HRT cycles may increase the risk for hypertensive disorders of pregnancy (HDP) [6]. Large retrospective cohort studies have shown FET of HRT cycles to be associated with a higher risk of HDP than FET of natural ovulatory [7] or letrozole-stimulated cycles [8]. In addition, long-term use of exogenous estrogen may increase the risk of thromboembolic events [9].

Letrozole, a third-generation aromatase inhibitor, reduces the production of estrogen via aromatase inhibition [10]. A hypoestrogenic state causes release of the hypothalamic-pituitary axis from estrogenic negative feedback, which in turn increases follicle-stimulating hormone (FSH) secretion and ovarian follicle development. Because letrozole does not antagonize estrogen receptors and maintains the normal central feedback, it generally leads to mono-ovulatory cycles, which can reduce the risk of OHSS [11]. In addition, letrozole has a short half-life of about 48 h, so estrogen-target tissues (such as the endometrium and cervix) are potentially spared adverse effects [11, 12]. Legro et al. found that as compared with clomiphene, letrozole was associated with higher live birth and ovulation rates among infertile women with PCOS [13]. In 2018, EBG recommended that letrozole should be considered first-line pharmacological treatment for ovulation induction in women with PCOS and anovulatory infertility [2]. However, letrozole is still rarely used in FET. To date, only a few retrospective studies and a small sample RCT have compared the effects of letrozole-stimulated (LS) and HRT protocols. Most of these studies were small [14–16] and only two showed live birth outcomes [14, 17]. Studies comparing the safety of the two protocols, especially in women with PCOS, are also scarce, and there is currently no large cohort study spanning from infertility treatment to delivery that comprehensively compares the effectiveness and safety of the two endometrial preparation protocols and provides conclusions regarding protocol superiority. In addition, patients with PCOS are heterogeneous and phenotypically diverse, and there is a lack of evidence on whether LS protocol is suitable for some specific phenotypes.

This study aims to comprehensively compare the effectiveness and safety (including pregnancy and obstetric outcomes) of HRT and LS in FET cycles performed in women with PCOS through a cohort, and explore the suitable population of LS, so as to provide evidence-based evidence for the selection of endometrial preparation protocol for women with PCOS.

## Methods

### Study population and design

The was a retrospective cohort study conducted at the Reproductive and.

Genetic Hospital of CITIC-Xiangya. All FET cycles performed in women with PCOS from January 2019 to December 2020 were reviewed for potential inclusion. The diagnosis of PCOS was based on modified Rotterdam criteria [18], i.e. menstrual abnormalities (irregular uterine bleeding, oligomenorrhea, or amenorrhea) with hyperandrogenism (hirsutism and/or hyperandrogenemia) and/or polycystic ovaries (at least an ovary containing 12 or more antral follicles of 2 to 9 mm in diameter and/or at least an ovary volume > 10 cm^3^). HRT and LS cycles with live birth outcome were included in the study. Participants were excluded if any of the following exclusion criteria were met: (1) use of preimplantation genetic test; (2) a history of recurrent spontaneous abortion; (3) presence of congenital uterine malformations; or (4) use of sequential embryo transfer.

### Endometrial preparation

In LS protocol, letrozole (2.5 mg: letrofome, Zhejiang Hisun Pharmaceutical Co., Ltd, Zhejiang, China) was administered for 5 consecutive days, during days 3 to 5 of a natural or progesterone(P)-induced menstrual cycle. Ultrasound monitoring and serum hormone measurement were performed from cycle days 12–13 onwards.

1. If the leading follicle reached a diameter of ≥ 12 mm on days 12–13, transvaginal ultrasound was repeated until ovulation. When the diameter of the dominant follicle was ≥ 18-20 mm without ovulation and the endometrium thickness was ≥ 8 mm, P was<1ng/ml, human chorionic gonadotropin ampules (HCG) 5000-10,000 IU was injected for final oocyte triggering. Luteal support (10 mg, twice daily; Duphaston, Abbott Biologicals B.V., Abbott Park, IL) was initiated on the day of ovulation and continued until 28 days after embryo transfer if a pregnancy occurred. For luteinized unruptured follicles, the day when the P level was ≥ 1.4ng/ml after 48 h of injecting HCG or the day when the follicular diameter was ≥ 18–20, urine luteinizing hormone (LH) was positive, and P was ≥ 1.4 ng/ml was assumed to be the ovulation day.
2. If the diameter of a dominant follicle was < 12 mm and P was < 1ng/ml on day 12–13, a daily dosage of 75 IU Human Menopausal Gonadotropin (HMG) (Livzon Pharmaceutical Group Inc.) was supplemented to stimulate follicle growth. If there was a dominant follicle after using HMG for 3–5 days, HMG was used continuously until the day of HCG; if there was still no dominant follicle but the thickness of the endometrium reached ≥ 8 mm and P was < 1ng/ml, it would be replaced with HRT protocol to transform the endometrium, and these cycles would be not included in the analysis. If there were more than 3 dominant follicles (follicle diameter ≥ 14 mm) or endometrial thickness was < 8 mm on the day before transfer, the LS cycle would be canceled.

In the HRT protocol, oral estradiol (E2) valerate tablet (3 mg, twice daily, Progynova, Bayer Schering Pharma, Berlin, Germany) administration was commenced on days 3–5 of a natural or P-induced menstrual cycle. Ten days later, the endometrial thickness reached ≥ 8 mm with P<1ng/ml, P vaginal suppositories (200 mg, three times daily; Utrogestan, Besins Healthcare, Paris, France) were given an oral Dydrogesterone Tablets (10 mg, twice daily; Duphaston, Abbott Biologicals B.V.) was initiated on the next day. If clinical pregnancy was confirmed, the E2 valerate dose was reduced to 4 mg per day, and the dose would be reduced again to 2 mg per day at 45 days after embryo transfer until the drug was discontinued at 55 days after embryo transfer. The combination of Utrogestan and Duphaston was used until 45 days after embryo transfer, and then Duphaston was used alone until 70 days after embryo transfer. If P was > 1ng/ml on the day of luteal support or endometrial thickness was < 8 mm on the day before transfer, the HRT cycle would be canceled.

### Embryo vitrification, thawing, and transfer

All embryos were graded before freezing. On day 3, embryos were scored using the Puissant’s criterion, and blastocysts were graded according to Gardner and Schoolcraft’s system on days 5/6/7. All embryos were vitrified and thawed using a Kitazato vitrification kit (Kitazato Biopharma, Shizuoka, Japan) in combination with closed High-Security Vitrification Straws (Cryo Bio System, France). Embryos were thawed on the day of transfer. Thawed embryos were prioritized based on best quality before freezing. The thawed embryos were then transferred to G2.5 medium and cultured for 2–6 h. Embryos were considered as survival and suitable for transfer when half or more of the blastomeres recovered or the blastocyst re-expanded.

Two cleavage-stage embryos, one good-quality blastocyst, or 1–2 non-good blastocysts (depending on the patient’s condition or wishes) were transferred. For LS protocol, the day of ovulation was defined as day 0, cleavage stage embryos were transferred on day 3, and blastocysts were transferred on day 5. For the HRT protocol, the day of starting progesterone supplementation was considered as P + 0, cleavage stage embryos were transferred on the day of P + 3, and blastocysts were transferred on the day of P + 5.

### Outcome measures

The primary outcome was live birth, defined as the delivery of a viable infant at 28 gestational weeks or more. The secondary outcomes included clinical pregnancy (presence of at least one gestational sac in the uterine cavity on ultrasound at approximately 28 days after embryo transfer), miscarriage (loss of clinical pregnancy before the 28th gestational week), early miscarriage (loss of clinical pregnancy before the 12th gestational week), hypertensive disorders of pregnancy (defined as the development of blood pressure > 140/90 mm Hg after pregnancy with or without proteinuria or other signs of preeclampsia, including preeclampsia and gestational hypertension and excludes chronic hypertension), gestational diabetes mellitus (GDM) (defined as carbohydrate intolerance of variable severity with onset or first recognition during pregnancy as determined from the diagnosis in the obstetrical medical record), preterm birth (delivery of a fetus at less than 37 and more than 28 weeks’ gestational age), stillbirth (death of a child born at a gestational age of ≥ 20 weeks or weighing ≥ 500 g), mode of delivery, small gestational age (SGA) (birthweight below the 10th percentile for gestational age), and large gestational age (LGA) (birthweight above the 90th percentile for gestational age). The reference of birthweight for gestational age and neonatal gender was based on the Chinese population [19, 20].

### Definitions of covariates

Good quality embryos included day 3 cleavage stage embryos with grade 7CI /8CI or day 5/6 blastocysts with grade 4BB or higher. Insulin resistance (IR) was diagnosed when at least one of the following five criteria were met: fasting insulin level > 20miu/m; homeostasis model assessment of insulin resistance (HOMA-IR) ≥ 2.69, which was simplified by the formula: fasting insulin (mU/dL) * fasting blood glucose (mmol/L)/22.5; insulin level at 1 h after a meal was 10 times higher than fasting insulin level; insulin levels at 2 h after a meal > insulin levels at 1 h after a meal; insulin level at 3 h after a meal did not fall to the fasting level (below 20miu/ml). Central obesity, elevation of fasting plasma glucose (FPG), and elevation of blood pressure were defined according to the diagnostic criteria for metabolic syndrome [21]. Central obesity was defined as waist circumference for Asian women ≥ 80 cm. Elevation of FPG was defined as FPG ≥ 5.6 mmol/L (100 mg/dL) or specific medication to treat. Elevation of blood pressure was defined as blood pressure ≥ 130/85 mm Hg or specific medication to treat. PCOS was subdivided into four phenotypes: phenotype A: hyperandrogenism + ovulatory dysfunction + polycystic ovarian morphology; phenotype B: hyperandrogenism + ovulatory dysfunction; phenotype C: hyperandrogenism + polycystic ovarian morphology; and phenotype D: ovulatory dysfunction + polycystic ovarian morphology [22].

### Statistical analysis

Continuous variables were represented as means ± standard deviation, and categorical variables were described as frequency and percentage. Differences between HRT and LS groups were evaluated by Mann-Whitney U-test for continuous variables and the chi-square test for categorical variables. The crude and adjusted odds ratios (cOR and aOR, respectively) of LS compared with HRT for pregnancy and obstetric outcomes were evaluated by logistic regression analysis. Factors with statistically significant differences in univariate analysis and those that had potential impact on outcomes (e.g. endometrial thickness before transfer, number of embryos transferred, quality of embryos transferred, developmental stage of embryos transferred and basic diseases of women (including moderate to severe intrauterine adhesions, untreated hydrosalpinx, adenomyosis, endometritis and scarred uterus)) were included in the multivariate models for adjustment. To further explore the population for which LS may be applicable, effects of LS on LBR were analyzed in the subgroup analysis. Lastly, interactions of the endometrial preparation protocols with the stratification factors were analyzed using binary logistic regression.

All statistical analyses were performed using IBM SPSS Statistics 24 (IBM Corp., Armonk, NY, US). Forest plots for subgroup analyses were drawn using R software (version 4.2.2). Significance tests were two-tailed and conducted at the 0.05 significance level.

## Results

A total of 3707 FET cycles in patients with PCOS were included in the analysis. Of these, 1700 cycles underwent HRT, and 2007 cycles underwent LS protocol. The basic characteristics of the two groups are shown in Table 1. The results of univariate analysis showed that there were statistically significant differences in maternal age, maternal body mass index (BMI), FSH, LH/FSH, anti-Mullerian hormone (AMH), antral follicle count (AFC), progesterone (P), and E2 before embryo transfer, developmental stage of embryos transferred, and IR between the two groups, and other characteristics were comparable (Table 1).

**Table 1:** Basic characteristics of HRT and LS groups

Characteristics	HRT group (n = 1700)	LS group (n = 2007)	P
Maternal age (years)	29.49 ± 3.58	29.86 ± 3.76	0.002
Maternal body mass index(kg/m^2^)	22.44 ± 2.91	22.27 ± 2.96	0.033
Infertility duration (years)	3.64 ± 2.44	3.61 ± 2.5	0.443
Gravidity	0.67 ± 1.03	0.68 ± 1.04	0.905
Parity	0.11 ± 0.34	0.12 ± 0.36	0.379
Cause of infertility			0.177
Male factor	453(29.53)	520(26.98)	
Endometriosis	22(1.43)	25(1.30)	
Uterine factor	258(16.82)	295(15.31)	
Tubal factor	718(46.81)	982(50.96)	
Unexplained infertility	83(5.41)	105(5.45)	
FSH (IU/L)	5.28 ± 1.41	5.47 ± 1.47	< 0.001
LH (IU/L)	7.35 ± 6.02	7.19 ± 5.25	0.403
LH/FSH	1.41 ± 1.06	1.34 ± 0.95	0.009
Total T (ng/mL)	1.47 ± 6.46	2.41 ± 9.17	0.055
AMH (ng/mL)	12.46 ± 6.69	11.13 ± 6.73	< 0.001
AFC	47.48 ± 24.6	48.82 ± 23.44	0.007
Menstrual cycle (days)	57.98 ± 36.08	53.92 ± 28.06	0.144
P before embryo transfer(ng/ml)	8.64 ± 3.87	13.64 ± 6.99	< 0.001
E2 before embryo transfer(pg/ml)	448.97 ± 674.36	110.79 ± 184.88	< 0.001
Endometrial thickness before transfer(mm)	11.58 ± 1.7	11.49 ± 1.64	0.353
Number of embryos transferred	1.55 ± 0.5	1.58 ± 0.49	0.097
Quality of embryos transferred			0.198
Good quality	1124(66.12)	1367(68.11)	
Non-good quality	576(33.88)	640(31.89)	
Developmental stage of embryos transferred			0.010
Blastocyst (day 5)	1126(66.24)	1247(62.13)	
Cleavage stage (day 3)	574(33.76)	760(37.87)	
Phenotypes of metabolic disorder			
IR	1355(79.71)	1694(84.4)	< 0.001
Elevation of fasting plasma glucose	212(15.33)	247(14.02)	0.301
Elevation of blood pressure	282(17)	320(16.14)	0.486
Central obesity	470(27.65)	509(25.36)	0.116

Date were expressed as the mean ± standard deviation or number (%)

Difference between the groups were analyzed by the Mann-Whitney U-test or chi-squared test

### Pregnancy and obstetric outcomes

The clinical pregnancy rate (CPR, 70.9% vs. 64.4%, *P*<0.001), LBR (60.5% vs. 51.4%, *P*<0.001), and incidence of SGA in singleton pregnancies (6.0% vs. 3.3%, *P* = 0.013) in the LS group were higher than in the HRT group. In contrast, the miscarriage rate (14.7% vs. 20.1%, *P*<0.001), early miscarriage rate (10.4% vs. 13.8%, *P* = 0.009), incidence of GDM (16.7% vs. 20.7%, *P* = 0.020), cesarean delivery (72.2% vs. 76.6%, *P* = 0.023), LGA (15.0% vs. 20.0%, *P* = 0.009) in singleton pregnancies, and LGA (10.6% vs. 15.3%, *P* = 0.035) in twin pregnancies were lower in the LS group than in the HRT group. The incidence of HDP, preterm birth, stillbirths, and the number of fetuses were comparable between the two groups (Table 2). Letrozole cycles were further divided into letrozole alone and letrozole + HMG subgroups. The incidence of SGA in singleton pregnancies was higher in the letrozole alone subgroup than in the letrozole + HMG subgroup, and other pregnancy and obstetric outcomes were comparable between the two subgroups (Additional Table 1).

**Table 2:** Pregnancy and obstetric outcomes of HRT and LS groups

Outcomes		HRT cycles (n = 1700)	LS cycles (n = 2007)	P
Clinical pregnancy rate		1095(64.4)	1423(70.9)	< 0.001
Miscarriage rate		220(20.1)	209(14.7)	< 0.001
Early miscarriage rate		151(13.8)	148(10.4)	0.009
Hypertensive disorders of pregnancy		78(8.9)	81(6.7)	0.056
Gestational diabetes mellitus		181(20.7)	203(16.7)	0.020
Live birth rate		874(51.4)	1214(60.5)	< 0.001
Gestational age (weeks)		37.41 ± 2.34	37.43 ± 1.99	0.175
Preterm birth		278(31.8)	390(32.1)	0.878
Stillbirth		1(0.1)	0(0.00)	0.254
Mode of delivery				0.023
Vaginal		204(23.4)	337(27.8)	
Cesarean delivery		668(76.6)	875(72.2)	
Number of fetuses				
1		701(80.2)	924(76.1)	0.048
2		173(19.8)	288(23.7)	
3 or more		0(0)	2(0.2)	
Birthweight				
Singleton		3311.6 ± 540.13	3271.7 ± 507.03	0.039
Twin		2440.26 ± 520.42	2511.23 ± 434.49	0.199
SGA				
Singleton		23(3.3)	55(6.0)	0.013
Twin		13(3.8)	22(3.8)	0.962
LGA				
Singleton		140(20.0)	139(15.0)	0.009
Twin		53(15.3)	61(10.6)	0.035

Date were expressed as the mean ± standard deviation or number (%). Difference between the groups were analyzed by the Mann-Whitney U-test or chi-squared test

After adjusting for possible confounding factors using multivariate logistics regression models, LS remained significantly associated with higher CPR (aOR, 1.41; 95% CI,1.18–1.68), LBR (aOR,1.49; 95% CI, 1.27–1.76) and a lower risk of miscarriage (aOR, 0.68; 95% CI, 0.53–0.89), early miscarriage (aOR, 0.69; 95% CI, 0.51–0.94), HDP (aOR, 0.63; 95% CI, 0.42–0.95), and GDM (aOR,0.71; 95% CI, 0.53–0.93) compared with HRT (Table 3).

**Table 3:** Crude and adjusted odds rations of pregnancy and obstetric outcomes of HRT and LS groups

Outcomes	cOR (95%CI)	aOR (95%CI)
Clinical pregnancy rate	**1.35(1.17,1.55)**	**1.41(1.18,1.68)** ^**a**^
Miscarriage rate	**0.68(0.56,0.84)**	**0.68(0.53,0.89)** ^**a**^
Early miscarriage rate	**0.73(0.57,0.92)**	**0.69(0.51,0.94)** ^**a**^
Hypertensive disorders of pregnancy	0.73(0.53,1.01)	**0.63(0.42,0.95)** ^**a**^
Gestational diabetes mellitus	**0.77(0.62,0.96)**	**0.71(0.53,0.93)** ^**a**^
Live birth rate	**1.45(1.27,1.65)**	**1.49(1.27,1.76)** ^**a**^
Preterm birth	0.99(0.82,1.19)	1.09(0.86,1.38)^b^
Cesarean delivery	**0.8(0.65,0.97)**	0.78(0.60,1.01)^c^
SGA		
Singleton	**1.87(1.14,3.07)**	1.58(0.81,3.05)^b^
Twin	1.02(0.51,2.05)	0.79(0.33,1.90)^b^
LGA		
Singleton	**0.71(0.55,0.92)**	0.81(0.58,1.13)^b^
Twin	**0.65(0.44,0.97)**	0.62(0.38,1.00)^b^

OR, odds ratio; CI, confidence interval. Odds ratios were obtained via a multiple logistic regression analysis. Significantly increased or reduced odds are indicated by boldface

a Adjusted for maternal age, maternal BMI, FSH, LH/FSH, AMH, AFC, P before embryo transfer, E2 before embryo transfer, endometrial thickness before transfer, number of embryos transferred, quality of embryos transferred, developmental stage of embryos transferred, IR and basic diseases of women

b Adjusted for maternal age, maternal BMI, FSH, LH/FSH, AMH, AFC, P before embryo transfer, E2 before embryo transfer, endometrial thickness before transfer, number of embryos transferred, quality of embryos transferred, developmental stage of embryos transferred, IR, basic diseases of women, HDP and GDM

c Adjusted for maternal age, maternal BMI, FSH, LH/FSH, AMH, AFC, P before embryo transfer, E2 before embryo transfer, endometrial thickness before transfer, number of embryos transferred, quality of embryos transferred, developmental stage of embryos transferred, IR, basic diseases of women, HDP, GDM and gestational age

### Subgroup analysis of live birth

**Fig. 1:**
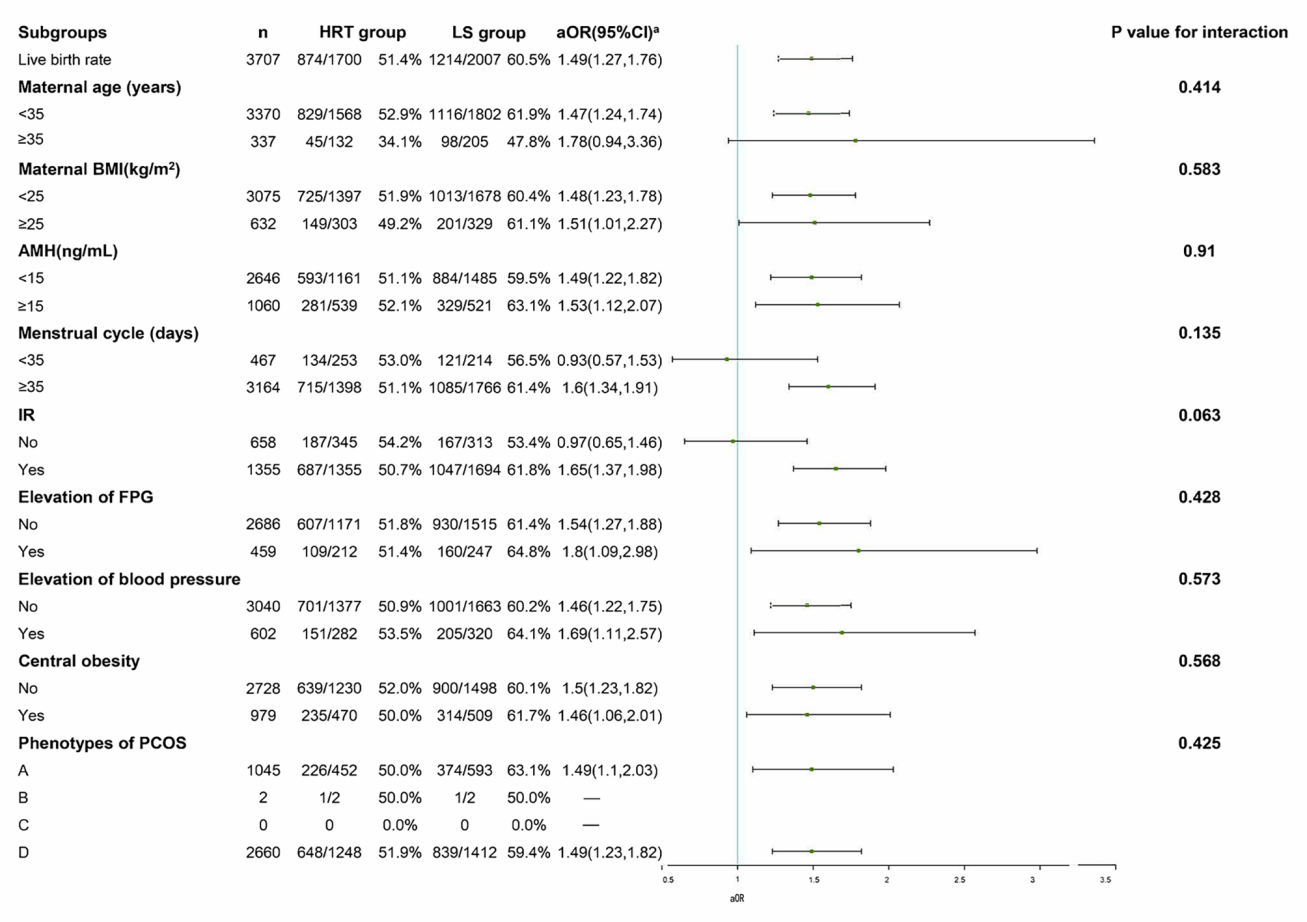
Subgroup analysis of live birth rate. **a** Adjusted factors including maternal age, maternal BMI, FSH, LH/FSH, AMH, AFC, P before embryo transfer, E2 before embryo transfer, endometrial thickness before transfer, number of embryos transferred, quality of embryos transferred, developmental stage of embryos transferred, IR and basic diseases of women. When a factor is used for subgroup analysis, it is no longer adjusted

Factors including maternal age, BMI, AMH, menstrual cycle, IR, elevation of FPG, elevation of blood pressure, central obesity, and phenotypes of PCOS were stratified and effects of LS on LBR were analyzed in the subgroup analysis. LS had higher LBRs than HRT in nearly all subgroups, except for the maternal age ≥ 35 years, menstrual cycle < 35 days, and no IR subgroups. There was no significant interaction found between these stratified factors and endometrial preparation protocols (Fig. 1).

## Discussion

This large retrospective cohort study found that LS improved CPR and LBR, reduced miscarriage rate, and lowered the risk of HDP and GDM compared with HRT in women with PCOS. The results of subgroup analysis showed that LS was appropriate for all types of patients with PCOS in terms of live birth outcome.

Currently, few studies exploring the efficacy of LS protocol in FET exist. A small number of retrospective studies and one small RCT previously compared pregnancy outcomes between LS with HRT. Several small studies by Aslih et al. [14] and Hu et al. [16] and Lee et al. [23] found that the CPRs of the LS were higher than that of HRT, with a combined OR of 1.54 (1.20, 1.98); miscarriage rates were lower than HRT, and no statistical differences were found [24]. Another study from Zhangjie et al. [17] was the largest thus far to compare pregnancy outcomes between and LS (n = 1571) and HRT (n = 1093); this was one of only two studies that compared the live birth outcome between the two protocols [14, 17]. Their results showed that after adjusting for confounders, there was no difference in CPRs between the two groups and that LS reduced the miscarriage rate and increased LBR. Our study had a large sample size and showed that the CPR and LBR in the LS group were significantly higher than in the HRT group; the LS group’s miscarriage rate was also lower than that of HRT, and the results were consistent before and after adjusting for confounding factors. Most pregnancy and obstetric outcomes were consistent with letrozole alone and letrozole + HMG subpopulations, suggesting that there may be differences in ovarian responsiveness to letrozole, but clinical outcomes were not affected. Possible explanations for our results differing slightly from previous studies include: (1) our subjects were patients with PCOS who met the modified Rotterdam criteria, all of whom had irregular menstrual cycles, thus these patients had potential ovulation abnormalities, while Zhang et al. [17] studied patients with PCOS who met the Rotterdam criteria, which meant that not all of them had ovulation abnormalities; (2) the time of ovulation in the LS group of this study was determined based on ultrasound/P, while the previous study used a fixed-time transfer following HCG trigger. The ovulation time in this study was relatively more accurate, which may be one of the reasons for the relatively higher CPR in this study compared with others [17]. Published literature has confirmed that patients with PCOS are sensitive to estrogen and have a higher need for P [13, 25]. Letrozole, as an aromatase inhibitor, may improve clinical outcomes by reducing E2 and increasing P in the luteal phase. Our study showed that the average level of E2 on the day before transfer in the LS group was indeed significantly lower than that in the HRT group, while P levels were significantly higher than those in the HRT group, which may be associated with improved pregnancy outcomes in the LS group. In addition, other previous studies have found that letrozole can positively influence a number of markers of endometrial receptivity, such as leukemia inhibitory factor (LIF), fibroblast growth factor 22, and endometrial mRNA expression of LIF/GP130 receptor (LIFR) [26–28], suggesting that letrozole may increase CPR and LBR by improving endometrial receptivity. In addition, subgroup analysis found that most subgroups of patients with PCOS may benefit from LS. There was no interaction effect found between endometrial preparation protocols and the stratified factors, such as age, AMH, or the like. We also looked at trends in OR values for each subgroup, taking into account that the post hoc subgroup analysis may have failed to detect statistical differences due to insufficient sample sizes in the subgroups. The benefit trend of LS appeared to be more pronounced in subgroups of patients with PCOS with prolonged menstrual cycles and IR than their opposite subgroups (OR of menstrual cycle ≥ 35 days: 1.6, 95% CI:1.34–1.91 VS OR of menstrual cycle < 35 days: 0.93, 95% CI:0.57–1.53; OR of IR:1.65, 95% CI: 1.37–1.98 VS OR of non-IR:0.97, 95% CI:0.65–1.46). A possible reason for LS being more helpful in improving live birth outcome in patients with PCOS with IR and the prolonged menstrual cycle was that letrozole also alleviated IR by inhibiting aromatase activity. One of the causes of IR and prolonged menstrual cycle in patients with PCOS is abnormal metabolism of aromatase P450 serine [29]. Excess insulin acts on the insulin receptor in the pituitary gland, which increases LH secretion and stimulates the ovaries and adrenal glands to secrete androgens. It also increases free androgens by combining with sex hormone-binding globulins secreted by the liver, thereby inhibiting the maturation and excretion of follicles, further increasing the prolongation of the menstrual cycle. Previous literature has also reported that IR reduces CPR and increases miscarriage rates [30]. Our data suggested that letrozole may improve pregnancy outcomes by improving IR; however, more evidence is needed.

There have been a few studies comparing the effects of endometrial preparation protocols on maternal complications and obstetric outcomes. The studies from Zhangjie et al. [8] and Wenjuan Zhang et al. [31] were the only papers to compare the effects of LS and HRT on maternal complications and obstetric outcomes in women with PCOS or ovulatory disorder. Both Zhangjie et al. and the present study have shown that LS can reduce the risk of HDP. Previous studies have also found that the HRT increased the risk of HDP relative to the natural or stimulation cycles [7, 32, 33]. Recent work has also indicated that the absence of corpus luteum may be a potential factor predisposing women to HDP [6, 33, 34]. The absence of the corpus luteum in HRT cycles leads to the absence of substances such as relaxin and vascular endothelial growth factor, which affects early placental development and leads to HDP [35–37]. Our findings showed a significantly lower risk of HDP in the LS group (OR: 0.63, 95%CI:0.42–0.95), consistent with previous findings.

A high proportion of patients with PCOS have abnormal glucose metabolism, such as IR [38]. Continued development of IR may lead to glucose intolerance or type II diabetes [39]. The insulin signaling defect is due to serine phosphorylation of insulin receptors and IRS-1 secondary to intracellular serine kinases in PCOS, which results in decreased insulin-mediated activation of PI3-K and resistance to the metabolic actions of insulin has been postulated that the same kinase may inhibit insulin signaling in PCOS [29]. Letrozole may alleviate IR and the development of glucose tolerance and type II diabetes by reducing the action of P450. Therefore, letrozole may theoretically reduce the risk of GDM in patients with PCOS by alleviating abnormal glucose metabolism. This may explain to some extent our finding that LS reduced the risk of GDM compared with HRT; however, the specific mechanism for this needs to be further studied. Previous studies on the effect of endometrial preparation protocols on GDM have been inconsistent [7, 8, 31, 40, 41]. This may be due to inconsistent endometrial preparation protocols such as letrozole, clomiphene, or HMG induction across studies; alternatively, the study populations may have differed, and previous studies were not specific to patients with PCOS with irregular menstrual cycles.

Previous studies have found that LS reduced the incidence of cesarean delivery [8, 41]. In our univariate analysis, we also found that the incidence of cesarean delivery was lower in the LS group, but after adjusting for confounding factors including scarred uterus, there was no difference in the risk of cesarean delivery between LS and HRT groups. As scarred uterus was an important factor affecting cesarean delivery, this may be responsible for the inconsistency in this outcome. Of course, this difference can also be caused by the occurrence of cesarean by subjective patient choice.

Our study has several strengths. First, using a large sample size, this study comprehensively analyzed the effects of HRT and LS on pregnancy outcomes and obstetric outcomes for FET in women with PCOS, which could effectively combine the effectiveness and safety to comprehensively evaluate the pros and cons of the LS protocol. Second, this was the first study to explore applicable populations for the LS protocol, which may better guide clinical application. One limitation is the retrospective design of this study, which carries inherent bias. Patients receiving HRT and LS were non-randomized (mainly according to the doctor preference and patient wishes), so some characteristics differed between the two groups. Although we performed multivariable analysis to adjust confounding factors, this adjustment along with some unmeasured factors that could not be adjusted may still have an impact on pregnancy and obstetric outcomes. In addition, limited by retrospective studies, we could only observe the association of endometrial preparation protocols with outcomes, and the specific mechanism still needs further interventional and experimental studies.

## Conclusions

In conclusion, this retrospective cohort study found that LS could increase CPR and LBR, and reduce the incidence of miscarriage, HDP, and GDM compared with HRT in women with PCOS. LS protocol is suitable for all types of patients with PCOS, especially those with prolonged menstrual cycles or IR. LS protocol is a superior option to HRT for FET in women with PCOS. Future RCTs and mechanistic studies are needed to validate our results.

### Electronic supplementary material

Below is the link to the electronic supplementary material.

Supplementary Material 1. **Additional files** Additional Table 1: Pregnancy and obstetric outcomes of letrozole alone and letrozole + HMG subgroups

## Data Availability

The data underlying this article will be shared on reasonable request to the corresponding author.

## Abbreviations

PCOS: Polycystic ovary syndrome
LS: Letrozole-stimulated
HRT: Hormone replacement treatment
HDP: Hypertensive disorders of pregnancy
GDM: Gestational diabetes mellitus
CPR: Clinical pregnancy rate
LBR: Live birth rate
BMI: Body mass index
FSH: Follicle-stimulating hormone
LH: Luteinizing hormone
AMH: Anti-Mullerian hormone
AFC: Antral follicle count
P: Progesterone
E2: Estradiol
cOR: Crude odds ratio
aOR: Adjusted odds ratio
CI: Confidence intervals
SGA: Small gestational age
LGA: Large gestational age
IR: Insulin resistance
FPG: Fasting plasma glucose
