# Supplementary material for: Letrozole-stimulated endometrial preparation protocol is a superior alternative to hormone replacement treatment for frozen embryo transfer in women with polycystic ovary syndrome, a cohort study
Source: Reprod Biol Endocrinol. 2023 Oct 27;21:101. doi: 10.1186/s12958-023-01154-x (PMC10605334; doi:10.1186/s12958-023-01154-x)
Supplement: Supplementary file 1 — Supplementary Material 1. Additional files Additional Table 1: Pregnancy and obstetric outcomes of letrozole alone and letrozole + HMG subgroups [file 12958_2023_1154_MOESM1_ESM.docx]

Additional Table 1 Pregnancy and obstetric outcomes of letrozole alone and letrozole+ HMG subgroups

| Outcomes |  | Letrozole alone  (n=1313) | Letrozole+ HMG  (n=694) | P |
| --- | --- | --- | --- | --- |
| Clinical pregnancy rate |  | 922(70.2) | 501(72.2) | 0.356 |
| Miscarriage rate |  | 130(14.1) | 79(15.8) | 0.396 |
| Early miscarriage rate |  | 97(10.5) | 51(10.2) | 0.841 |
| Hypertensive disorders of pregnancy |  | 52(6.6) | 29(6.9) | 0.839 |
| Gestational diabetes mellitus |  | 138(17.4) | 65(15.4) | 0.369 |
| Live birth rate |  | 792(60.3) | 422(60.8) | 0.832 |
| Gestational age (weeks) |  | 37.45±1.99 | 37.38±1.98 | 0.538 |
| Preterm birth |  | 247(31.2) | 143(33.9) | 0.337 |
| Stillbirth |  |  |  |  |
| Mode of delivery |  |  |  | 0.127 |
| Vaginal |  | 231(29.2) | 106(25.1) |  |
| Cesarean delivery |  | 559(70.8) | 316(74.9) |  |
| Number of fetuses |  |  |  | 0.893 |
| 1 |  | 602(76) | 322(76.3) |  |
| 2 |  | 189(23.9) | 99(23.5) |  |
| 3 or more |  | 1(0.1) | 1(0.2) |  |
| Birthweight |  |  |  |  |
| Singleton |  | 3261.28±510.53 | 3291.22±500.61 | 0.394 |
| Twin |  | 2510.71±434.18 | 2512.22±436.16 | 0.968 |
| SGA |  |  |  |  |
| Singleton |  | 43(7.1) | 12(3.7) | 0.037 |
| Twin |  | 14(3.7) | 8(4) | 0.841 |
| LGA |  |  |  |  |
| Singleton |  | 88(14.6) | 51(15.8) | 0.621 |
| Twin |  | 37(9.8) | 24(12.1) | 0.387 |

Date were expressed as the mean ± standard deviation or number (%).

Difference between the groups were analyzed by the Mann-Whitney U-test or chi-squared test.
